# Predictors of stroke favorable functional outcome in Guinea, results from the Conakry stroke registry

**DOI:** 10.1038/s41598-022-05057-6

**Published:** 2022-01-21

**Authors:** Fode Abass Cisse, Noémie Ligot, Kaba Conde, Djigué Souleymane Barry, Lamine Mohamed Toure, Mamadi Konate, Mohamed Fode Soumah, Karinka Diawara, Mohamed Traore, Gilles Naeije

**Affiliations:** 1 Department of Neurology, CHU Ignace Deen, Université Gamal Abdel Nasser Conakry (UGANC), Conakry, Guinea; 2 Department of Neurology, CUB Hôpital Erasme, Université Libre de Bruxelles (ULB), Brussels, Belgium; 3 Laboratoire de Cartographie Fonctionnelle du Cerveau, ULB Neuroscience Institute, Université Libre de Bruxelles (ULB), 808 Lennik Street, 1070 Brussels, Belgium

**Keywords:** Neurology, Neurological disorders, Stroke

## Abstract

Low- to middle-income countries (LMICs) now bear most of the stroke burden. In LMICs, stroke epidemiology and health care systems are different from HICs. Therefore, a high-income country (HIC)-based predictive model may not correspond to the LMIC stroke context. Identify the impact of modifiable variables in acute stroke management in Conakry, Guinea as potential predictors of favorable stroke outcome. Data were extracted from the Conakry stroke registry that includes 1018 patients. A logistic regression model was built to predict favorable stroke outcomes, defined as mRS 0–2. Age, admission NIHSS score, mean arterial blood pressure and capillary glycemia were chosen as covariates. Delay to brain CT imaging under 24 h from symptom onset, fever, presence of sores and abnormal lung auscultation were included as factors. NIHSS score on admission, age and ischemic stroke were included in the null model as nuisance parameters to determine the contribution of modifiable variables to predict stroke favorable outcome. Lower admission NIHSS, brain CT imaging within 24 h of symptoms onset and lower mean arterial blood pressure emerged as a significant positive predictors of favorable stroke outcome with respective odd ratios (OR) of 1.35 [1.28–1.43], 2.1 [1.16–3.8] and 1.01 [1.01–1.04]. The presence of fever or sores impacted negatively stroke favorable outcomes with OR of 0.3 [0.1–0.85] and 0.25 [0.14–0.45]. The area under receiver operating characteristic curves (AUC) of the model was 0.86. This model explained 44.5% of the variability of the favorable stroke outcome with 10.2% of the variability explained by the modifiable variables when admission NIHSS, and ischemic stroke were included in the null model as nuisance parameter. In the Conakry stroke registry, using a logistic regression to predict stroke favorable outcome, five variables that led to an AUC of 0.86: admission NIHSS, early brain CT imaging, fever, sores and mean blood pressure. This paves the way for future public health interventions to test whether modulating amendable variables leads to increased favorable stroke outcomes in LMICs.

## Introduction

Stroke is a leading cause of death and disability worldwide^1^. The burden of stroke has moved from high-income countries (HICs) to low-to-middle-income countries (LMICs). LMICs now host the heaviest part of the global stroke burden in terms of disability and death^2–4^. In many LMICs, acute brain imaging facilities, recanalization therapies and stroke treatment referral centers are neither accessible nor affordable for the vast majority of the population^5^. Most efforts to alleviate stroke burden are based on primary prevention^6,7^. However, pragmatic interventions within hospital stroke care management could help improve stroke outcome^8^, but the identification of potential targets for effective interventions still needs to be accurately singled out.

Many prediction models have been proposed over the years to isolate factors associated with favorable stroke outcomes^9^. However, the generalizability of such models remains controversial, as no single model is likely to address all situations, subgroups and local contexts. In particular, the bulk of models are validated in HIC and do not reflect LMIC stroke epidemiology and systems of care^10^. In HIC, models using clinical variables such as the six simple variables (SSV) that included age, the verbal component of the Glasgow Coma Scale (GCS), arm power, ability to walk, prestroke living condition and prestroke dependency were found to be efficient in predicting independent survival after stroke^11,12^. The addition of imaging data such as magnetic resonance diffusion-weighted imaging stroke volume^13^ or brain computed tomography characteristics to clinical data does not seem to improve prediction accuracy^14^. Interestingly, the accuracy of those models fails to outperform basic models that include only age and initial stroke severity based on the National Institute of Health Stroke Scale (NIHSS)^12,15^. These seminal models focused on intrinsic patient stroke characteristics that cannot be modified by medical management. Several models also show that amendable variable in acute stroke management such as the time to brain imaging, fever^16^, abnormal glycemia^17^, inflammation^18^, blood pressure levels^19,20^ can impact stroke outcome and could be the target of dedicated medical intervention in limited resource settings to improve stroke prognosis^8,21,61^. Similarly, sores and inhalation bronchopneumonia are major stroke medical complications that may be prevented and/or diagnosed early on clinical grounds to decrease stroke-associated morbi-mortality^22^.

The republic of Guinea is among the poorest countries in the world, with a medical doctor density of 7/100,000 inhabitants. The National Healthcare system is pyramidal, with the three national hospitals (Donka, Ignace Deen and Sino-Guinean) all located in the capital, Conakry, of which only Sino-Guinean is equipped with brain computer tomography (CT). The mean age of stroke onset is sixty years old, with a quarter of total stroke affecting patients younger than fifty years old reflecting a stroke epidemiology similar to many LMICs^6,21^.

Here, we aimed at determining the impact of potentially amendable variable in acute stroke diagnostic, management and care in a limited resource setting in Conakry, Guinea using a logistic regression model. The identification of amendable variables predictive of favorable stroke outcome in a limited resource setting, if any, could help designing interventions that may alleviate stroke burden in the future.

## Results

### Population (Table 1)

**Table 1:** Study patients’ characteristics.

	n	
**Epidemiological characteristics**		
Age, years (mean ± SD)	1014	59.5 ± 14.9
High blood pressure (%)	1018	68.1
Diabete (%)	1018	12.1
CMI (%)	1018	10.3
Alcohol (%)	1018	5.1
Smoking (%)	1018	6.6
HIV (%)	1018	1.9
**Stroke characteristics**		
NIHSS (mean ± SD)	1016	9.5 ± 5.3
Ischemic stroke (%)	1018	74.3
Intraparenchymal Hemorrage (%)	1018	20.0
**Amendable variables**		
CT within 24 h symptoms onset (%)	1018	9.5
Mean arterial blood pressure (mean ± SD)	1018	113.5 ± 33.4
Fever (%)	1018	16.7
Sores (%)	1018	24.3
Abnormal lung auscultation (%)	1018	6.3
Capillary glycemia	1017	148.1 ± 30.7
**Stroke outcome**		
mRS ≤ 2 (%)	784	23
mRS 3–5 (%)	784	70.3
mRS 6 (%)	784	6.8

Data from 1018 patients were extracted from the Conakry registry. Mean age was 59.5 ± 14.9 years. Mean admission NIHSS was 9.5 ± 5.3. Only 23% of patients experienced a favorable stroke outcome. The rest of epidemiological and clinical characteristics are summarized in Table 1.

### Logistic regression model (Fig. 1 illustrates the amendable variables associated to favorable stroke outcome)

**Figure 1:**
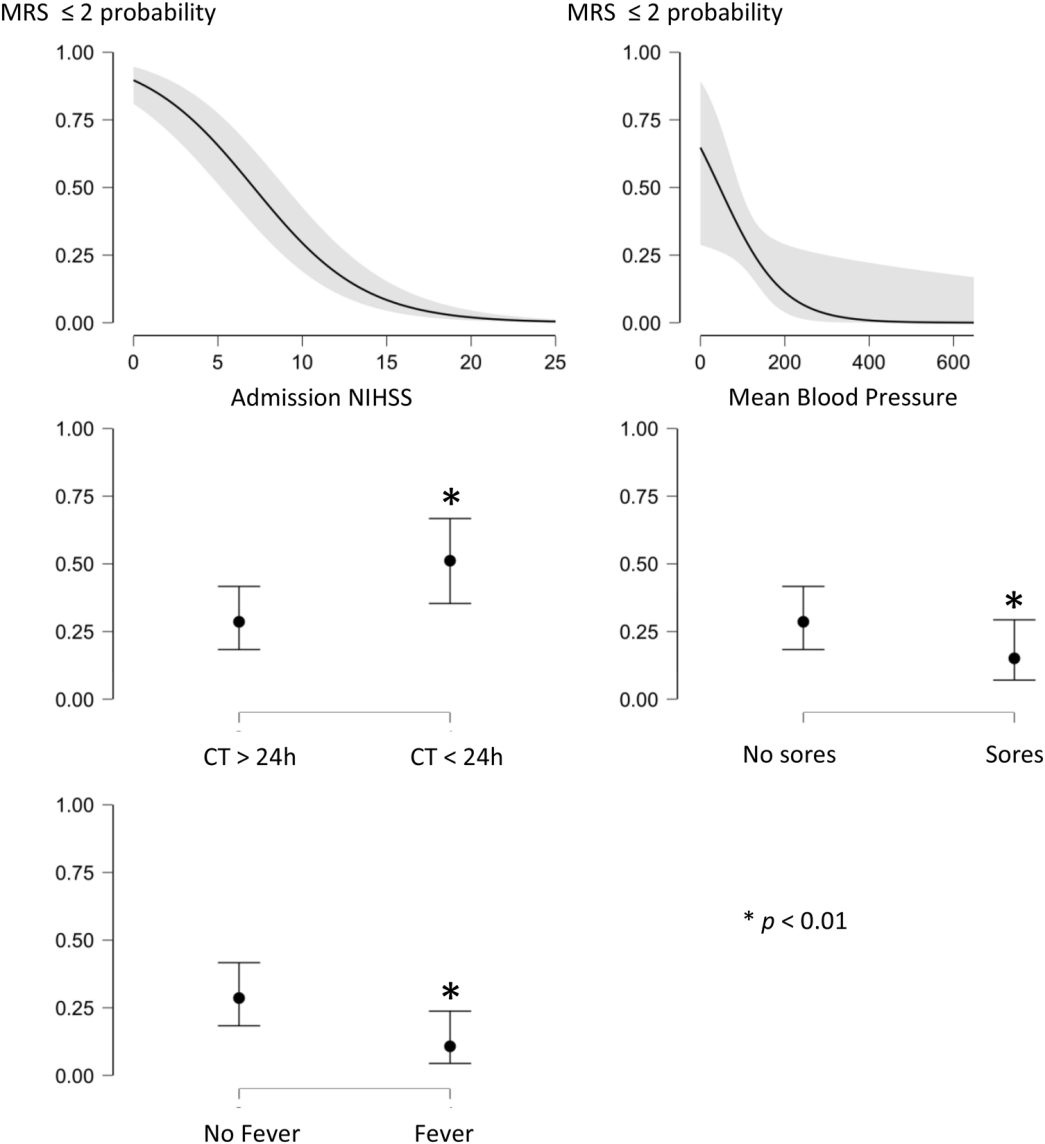
Probability of favorable outcome according to significant predictive variables. *MRS* modified Rankin Scale, *CT* brain computed tomography, *NIHSS* National institute of health stroke scale. The “y” axis of the graph corresponds to the probability of achieving a favorable outcome (0: no probability, 1: 100% of probability).

A logistic regression was performed to ascertain the effects of amendable variables on the likelihood that participants have a favorable stroke outcome (mRS ≤ 2).

Table 2 shows the respective Odd Ratios and associated *p* values for the variables assessed in the prediction model.

**Table 2:** Odd ratios of the variables assessed in the prediction model for a favorable stroke outcome (mRS ≤ 2).

	Odds ratio, [95% IC]	*p*
Lower NIHSS on admission	1.35 [1.28–1.43]	< 0.001
Younger age (years)	0.99 [0.98–1.01]	0.247
Ischemic stroke	2.0 [1.02–4]	0.044
CT within 24 h of symptoms onset	2.55 [1.11–5.8]	0.028
Lower mean arterial blood pressure	1.01 [1.01–1.04]	0.027
Lower capillary glycemia	1.21 [0.78–1.86]	0.47
Presence of scores	0.26 [0.15–0.45]	0.002
Presence of fever	0.3 [0.1–0.85]	0.025
Abnormal pulmonary auscultation	0.28 [0.03–2.67]	0.270

This logistic regression model was statistically significant to predict favorable outcomes (χ^2^ (degrees of freedom: 769) = 45.35, p < 0.001). The model AUC was 0.87 with a sensibility of 50% and a specificity of 95%.

A logistic regression performed using only variables significantly associated with favorable stroke outcome was also statistically significant (χ^2^ (775) = 44.93, p < 0.001). The AUC of this model was 0.86, with a sensitivity of 51.1% and specificity of 94.4%. Table 3 shows the respective odds ratios of this model.

**Table 3:** Odd ratios associated to significant variables for favorable stroke outcome (mRS ≤ 2).

	Odds ratio, [95% IC]	*p*
Lower NIHSS admission	1.33 [1.28–1.43]	< 0.001
Ischemic stroke	1.96 [1.03–3.85]	< 0.001
CT within 24 h of symptoms onset	2.1 [1.16–3.8]	0.014
Lower mean arterial blood pressure	1.01 [1.01–1.04]	0.017
Presence of scores	0.25 [0.14–0.45]	< 0.001
Presence of fever	0.3 [0.1–0.86]	0.019

This model explains 44.5% of the variability of the favorable stroke outcome (Nagelkerke determination coefficient, R^2^ = 0.445) with 10.2% (R^2^ 0.102) of the variability explained by the amendable variables when NIHSS on admission and ischemic stroke are included in the *null model* as nuisance parameters^23^. Without amendable variables, the AUC falls to 0.81 with a sensibility 42.2% and specificity of 95%.

## Discussion

This study used the Conakry stroke registry that includes over a thousand patients to identify four potentially modifiable variables: early brain CT imaging, fever, sores and mean blood pressure in stroke hospital care that are associated with favorable stroke outcome (mRS ≤ 2) in Guinea, a LMIC country in Sub-Saharan Africa.

Those results from the Conakry stroke registry are likely to be generalizable in similar settings in Sub-Saharan Africa (SSA). Indeed, our cohort matches the characteristics of prior reports in SSA in terms of age, sex, high blood pressure and diabetes prevalence, and ischemic stroke proportion^23–25^. Similarly, the mortality rate in Conakry stroke patients falls within the lower range of the rates reported in the InterStroke study^6^, which pooled data from Mozambique, Nigeria, South Africa and Sudan and the rates from smaller series in Cameroon^24^ and Congo^26,27^. This suggests that our population is representative of SSA LMIC stroke epidemiology. The stroke severity of our cohort, reflected by a mean NIHSS of 9.5, is four points lower than in the main stroke therapeutic trials on thrombolysis and thrombectomy in HIC^28,29^ but corresponds to the mean NIHSS levels from both SSA series^26,27,30,31^ and HIC cohorts on which stroke outcome predictive models were built^12,15,32^. The discrepancy between NIHSS levels in stroke therapeutic trials in HICs and the NIHSS levels in LMICs could be explained by a selection bias for more severe cases in therapeutic trials or by the fact that most severe cases in LMICs may fail to reach the hospital due to the lack of hospital accessibility in terms of distance, cost and medical transportation means^8^.

Our study shows that several predictors of favorable stroke outcome are different in the Guinean context than in models realized in HIC where patient populations and stroke care management are rather homogenous. Initially, we expected that admission NIHSS and patient age would be the main intrinsic predictive factors of favorable outcome. Indeed, the combination of admission NIHSS and age was found to be highly predictive of favorable stroke outcome in several studies, outperforming other clinical or imaging-based scores in HIC^12,14^. In our population, we found that age failed to predict better outcomes than HIC-based models, where age is consistently associated with outcome prediction^33–35^. This specificity in our population is likely because our population is almost fifteen years younger than in the HIC stroke studied populations^28,29,33–35^, with over a quarter of the patients who experience stroke before fifty years old in Guinea^21^. This age gap between stroke patients in HIC and Guinea and the overrepresentation of stroke in young individuals is not specific to Guinea but reflects the situation described in other African LMICs^6,36^. This suggests that age helps to predict favorable stroke outcome only in populations where stroke age distribution has a median age of approximately seventy, which is not the case in most LMICs^37,38^. On the other hand, admission NIHSS, expectedly, proved to be as important in our context than in HIC to predict favorable outcomes and was thus included in the *null model* of the logistic regression as a nuisance parameter^13,15,33^.

One major predictive factor for favorable outcome in our study was the realization of brain CT imaging within 24 h of symptom onset. Early brain imaging displayed odds ratios that exceeded two for favorable stroke outcome, which parallels the odds ratios found in seminal HIC studies^13^. While this may seem obvious in HIC, where reducing the delay to brain imaging is core to stroke management interventions^39^, it is challenging to implement in contexts such as Conakry. Indeed, brain CT is not available in the teaching hospital where the neurology ward stands. Patients have to be transported by their own means to surrounding private facilities and pay for the exam, a fee that corresponds to the mean annual Guinean income. Furthermore, as the brain CT facilities can only be found in the Conakry, a patient having a stroke in, for instance, Nzerekore that is at another border of the country, faces a journey of over 800 km to reach a brain CT facility. This situation, common to many LMIC African countries, also explains part of the long delay between stroke onset and hospital admission, which exceeds 90 h in Guinea^40^ and underlines the necessity to develop more accessible diagnostic centers to curb the stroke epidemic.

The occurrence of pressure sores negatively impacted the probability of favorable functional outcome, even after controlling in the model for admission NIHSS, suggesting a relation partly independent from stroke severity. The 25% prevalence of pressure sores in our cohort exceeds the prevalence usually reported in HIC stroke units, which is comprised between 2.5 and 21%^41,42^ but falls within the range described for patients in acute settings^43^ and patients hospitalized in similar SSA settings^44^. The prevalence of pressure sores is generally considered to reflect the health facility quality of care. The high prevalence of pressure sores in our study is probably related to the limited nurses resources, the lack of appropriates matrasses and the fact that family members are needed to assist in nursing care, a strategy shown ineffective to prevent pressure sores^45^. Dedicated intervention to improve both patient positioning and more regular position changes could, in our context, be an accessible approach to increase the likelihood of favorable stroke outcome.

Fever occurs in between 40 and 60% of stroke patients^46^ and worsens outcomes from stroke and brain injuries^47,48^. This association was also significant in our model highlighting the importance of fever symptomatic treatment in both HIC and LMIC settings. Inhalation pneumonia is classically sought for and treated on clinical grounds after stroke^49^ but abnormal lung auscultation was not a clear predictor of favorable outcome in our study. This fact hints either to other infections sites or to different mechanisms to accounts for the fever and its relationship with favorable stroke outcome in our model.

Finally, there was an inverse relation between mean arterial blood pressure and the likelihood of favorable stroke outcome reflecting findings from studies led in HIC^19,20,50^. However, the translation to that finding in medical intervention remains elusive. Some studies found that lowering blood pressure in stroke acute phase was either detrimental^51,52^ or ineffective^53^. Even if our context is different, those data from HIC warrant caution before trying to lower blood pressure in our stroke population and would require carefully designed study to address the issue without exposing patients to further risks of deterioration.

Our study is limited by several factors. First, mRS at hospital discharge as an outcome measure may not be a reliable surrogate for the 90-day mRS usually used in HIC predictive models of stroke outcomes. Even if there is a strong correlation between mRS at hospital discharge, 30-day mRS and 90-day mRS, this choice may have biased the results^52,53^. Second, the local context prevented extensive ancillary investigations or even the full stroke work-up to determine stroke etiology in most patients. Therefore, inflammatory markers, stroke subtypes, and eventual cardiac structural anomalies could not be included in the predictive model despite the described association between those factors and stroke functional outcomes^18,54,62^. Finally, despite the large cohort and the fact that the clinical characteristics of our population meet those of other cohorts in SSA, it is possible, even if unlikely, that our results correspond to a local snapshot and may be generalizable to other similar contexts in SSA.

In summary, our study in a large cohort of stroke patients from the Conakry stroke registry identified a predictive model for favorable stroke outcomes with an AUC similar to predictive models in HIC^12,15,33^. There were four modifiable variables: early brain CT imaging, fever, sores and mean blood pressure increased the proportion of stroke favorable outcome variability explained by the model beyond admission NIHSS. This study paves the way for medical and public health interventions that will test whether correcting those variables leads to increased favorable stroke outcomes in LMIC settings.

## Methods

### Population

The studied population is issued from a population-based stroke register in Conakry, Ignace Deen teaching hospital initiated in 2015^24^. This registry is based on the World Health Organization (WHO) definitions and stepwise approach to stroke surveillance (STEPS). WHO’s STEPS-1 focuses on patients admitted to an hospital with a stroke and helps identify health facility resources allocated to stroke patients and functional status of stroke patients at hospital discharge^55^.

### Ethics

All the experiment protocol for involving human data was in accordance with the Declaration of Helsinki. The local Ethics Committee of Ignace Deen Hospital (Comité National d'Ethique pour la Recherche en Santé, CNERS) approved the study, but waived the need for informed consent as only anonymous and operational monitoring data were collected and analyzed.

### Variable definition and logistic regression model

Stroke was defined according to the WHO definition^56^. Stroke severity was graded with the NIHSS and stroke outcome was based on the modified Rankin Scale (mRS)^57^ at hospital discharge^54,58^. Favorable stroke outcome was defined as mRS 0–2^59^.

The following amendable variables: fever^16^, abnormal glycemia^17^, blood pressure levels^18–20^ were selected based on their known association with stroke outcome, their diagnosis reliability using clinical examination or basic blood samples analysis in limited resource settings^8,21^.

Sores and abnormal pulmonary auscultation were used a markers of potentially preventable stroke complications due to the high prevalence of sores in patients hospitalized in sub-Saharan Africa^44^ and/or after stroke^42^ and the impact of inhalation bronchopneumonia on stroke mortality^22^.

Age in years, NIHSS, mean arterial blood pressure computed as in^50,60^ and capillary glycemia were chosen as covariates. Delay to brain CT imaging under 24 h from symptoms onset, fever defined as armpit temperature above 38 °C, presence of sores and abnormal lung auscultation were included as factors in a logistic regression model to predict stroke favorable outcome. Model ability to predict favorable stroke outcomes were assessed by calculating the area under receiver operating characteristic curves (AUC) of sensitivity versus (1-specificity). AUC is used to measure how well a model correctly classifies patients into two groups of favorable outcome or not. An AUC of 0.5 corresponds to a prediction that is no better than chance where 50% of patients would be assigned to each group, a higher AUC means better classification, and an AUC of 1 corresponds to perfect classification.

To assess the relative weight of amendable variables in the model, admission NIHSS and age that are unmodifiable intrinsic patients stroke characteristics were included in the *null model* as nuisance parameters.

## Data Availability

Data can be shared upon reasonable request.
